# Supplementary material for: HIVprotI: an integrated web based platform for prediction and design of HIV proteins inhibitors
Source: J Cheminform. 2018 Mar 9;10:12. doi: 10.1186/s13321-018-0266-y (PMC5845081; doi:10.1186/s13321-018-0266-y)
Supplement: Supplementary file 2 — Additional file 2. Source code of HIVProtI web server. [file 13321_2018_266_MOESM2_ESM.zip › HIVprotI_Source-code/hivprotI/weka-3-6-6/doc/weka/experiment/PairedCorrectedTTester.html]

PairedCorrectedTTester


---


|  |  |  |
| --- | --- | --- |
| **Overview** \| **Package** \| **Class** \| **Tree** \| **Deprecated** \| **Index** \| **Help** \| **Weka's home** | |  |
| **PREV CLASS**   **NEXT CLASS** | **FRAMES**    **NO FRAMES**     **All Classes** |  |
| SUMMARY: NESTED \| FIELD \| CONSTR \| METHOD | DETAIL: FIELD \| CONSTR \| METHOD |  |


---


## weka.experiment Class PairedCorrectedTTester

```
java.lang.Object
  weka.experiment.PairedTTester
      weka.experiment.PairedCorrectedTTester
```

**All Implemented Interfaces:**: java.io.Serializable, OptionHandler, RevisionHandler, TechnicalInformationHandler, Tester

---

``` public class PairedCorrectedTTester extends PairedTTester implements TechnicalInformationHandler ```

Behaves the same as PairedTTester, only it uses the corrected
resampled t-test statistic.

For more information see:


Claude Nadeau, Yoshua Bengio (2001). Inference for the Generalization Error. Machine Learning..


BibTeX:

```
 @article{Nadeau2001,
    author = {Claude Nadeau and Yoshua Bengio},
    journal = {Machine Learning},
    title = {Inference for the Generalization Error},
    year = {2001},
    PDF = {http://www.iro.umontreal.ca/\~lisa/bib/pub_subject/comparative/pointeurs/nadeau_MLJ1597.pdf}
 }
```


Valid options are:

```
 -D <index,index2-index4,...>
  Specify list of columns that specify a unique
  dataset.
  First and last are valid indexes. (default none)
```

```
 -R <index>
  Set the index of the column containing the run number
```

```
 -F <index>
  Set the index of the column containing the fold number
```

```
 -G <index1,index2-index4,...>
  Specify list of columns that specify a unique
  'result generator' (eg: classifier name and options).
  First and last are valid indexes. (default none)
```

```
 -S <significance level>
  Set the significance level for comparisons (default 0.05)
```

```
 -V
  Show standard deviations
```

```
 -L
  Produce table comparisons in Latex table format
```

```
 -csv
  Produce table comparisons in CSV table format
```

```
 -html
  Produce table comparisons in HTML table format
```

```
 -significance
  Produce table comparisons with only the significance values
```

```
 -gnuplot
  Produce table comparisons output suitable for GNUPlot
```

**Version:**
:   $Revision: 1.13 $

**Author:**
:   Richard Kirkby (rkirkby@cs.waikato.ac.nz)

**See Also:**: Serialized Form

---

**Constructor Summary**

`PairedCorrectedTTester()`


| **Method Summary** | |
| --- | --- |
| `PairedStats` | `calculateStatistics(Instance datasetSpecifier, int resultset1Index, int resultset2Index, int comparisonColumn)`             Computes a paired t-test comparison for a specified dataset between two resultsets. |
| `java.lang.String` | `getDisplayName()`             returns the name of the tester |
| `java.lang.String` | `getRevision()`             Returns the revision string. |
| `TechnicalInformation` | `getTechnicalInformation()`             Returns an instance of a TechnicalInformation object, containing detailed information about the technical background of this class, e.g., paper reference or book this class is based on. |
| `java.lang.String` | `getToolTipText()`             returns a string that is displayed as tooltip on the "perform test" button in the experimenter |
| `static void` | `main(java.lang.String[] args)`             Test the class from the command line. |

**Methods inherited from class weka.experiment.PairedTTester**

`assign, displayResultset, getDatasetKeyColumns, getDisplayedResultsets, getFoldColumn, getInstances, getNumDatasets, getNumResultsets, getOptions, getResultMatrix, getResultsetKeyColumns, getResultsetName, getRunColumn, getShowStdDevs, getSignificanceLevel, getSortColumn, getSortColumnName, header, listOptions, multiResultsetFull, multiResultsetRanking, multiResultsetSummary, multiResultsetWins, resultsetKey, setDatasetKeyColumns, setDisplayedResultsets, setFoldColumn, setInstances, setOptions, setResultMatrix, setResultsetKeyColumns, setRunColumn, setShowStdDevs, setSignificanceLevel, setSortColumn`

**Methods inherited from class java.lang.Object**

`equals, getClass, hashCode, notify, notifyAll, toString, wait, wait, wait`

**Constructor Detail**

### PairedCorrectedTTester

```
public PairedCorrectedTTester()
```


**Method Detail**

### getTechnicalInformation

```
public TechnicalInformation getTechnicalInformation()
```

:   Returns an instance of a TechnicalInformation object, containing
    detailed information about the technical background of this class,
    e.g., paper reference or book this class is based on.

    :   **Specified by:**: `getTechnicalInformation` in interface `TechnicalInformationHandler`
    :   **Returns:**: the technical information about this class

---


### calculateStatistics

```
public PairedStats calculateStatistics(Instance datasetSpecifier,
                                       int resultset1Index,
                                       int resultset2Index,
                                       int comparisonColumn)
                                throws java.lang.Exception
```

:   Computes a paired t-test comparison for a specified dataset between
    two resultsets.

    :   **Specified by:**: `calculateStatistics` in interface `Tester` **Overrides:**: `calculateStatistics` in class `PairedTTester`
    :   **Parameters:**: `datasetSpecifier` - the dataset specifier: `resultset1Index` - the index of the first resultset: `resultset2Index` - the index of the second resultset: `comparisonColumn` - the column containing values to compare **Returns:**: the results of the paired comparison **Throws:**: `java.lang.Exception` - if an error occurs

---


### main

```
public static void main(java.lang.String[] args)
```

:   Test the class from the command line.

    :   **Parameters:**: `args` - contains options for the instance ttests

---


### getDisplayName

```
public java.lang.String getDisplayName()
```

:   returns the name of the tester

    :   **Specified by:**: `getDisplayName` in interface `Tester` **Overrides:**: `getDisplayName` in class `PairedTTester`
    :   **Returns:**: the display name

---


### getToolTipText

```
public java.lang.String getToolTipText()
```

:   returns a string that is displayed as tooltip on the "perform test"
    button in the experimenter

    :   **Specified by:**: `getToolTipText` in interface `Tester` **Overrides:**: `getToolTipText` in class `PairedTTester`
    :   **Returns:**: the string for the tool tip

---


### getRevision

```
public java.lang.String getRevision()
```

:   Returns the revision string.

    :   **Specified by:**: `getRevision` in interface `RevisionHandler` **Overrides:**: `getRevision` in class `PairedTTester`
    :   **Returns:**: the revision


---


|  |  |  |
| --- | --- | --- |
| **Overview** \| **Package** \| **Class** \| **Tree** \| **Deprecated** \| **Index** \| **Help** \| **Weka's home** | |  |
| **PREV CLASS**   **NEXT CLASS** | **FRAMES**    **NO FRAMES**     **All Classes** |  |
| SUMMARY: NESTED \| FIELD \| CONSTR \| METHOD | DETAIL: FIELD \| CONSTR \| METHOD |  |


---
